# Hepatoprotective Effects of *Vernonia amygdalina* (Astereaceae) Extract on CCl_4_-Induced Liver Injury in Broiler Chickens

**DOI:** 10.3390/ani11123371

**Published:** 2021-11-25

**Authors:** Bemela Mawulom Tokofai, Kokou Idoh, Oyegunle Emmanuel Oke, Amegnona Agbonon

**Affiliations:** 1 Laboratoire des Techniques de Production Avicole, Centre d’Excellence Régional sur les Sciences Aviaires (CERSA), Université de Lomé, Lome 1515, Togo; emaoke7@yahoo.co.uk; 2 Laboratoire de Physiologie et de Pharmacologie, Faculté des Sciences, Université de Lomé, Lome 1515, Togo; idohkokou@gmail.com (K.I.); aamegnona3@gmail.com (A.A.); 3 Department of Animal Physiology, Federal University of Agriculture Abeokuta, Abeokuta 2240, Nigeria

**Keywords:** *Vernonia amygdalina*, CCl_4_, oxidative stress, hepatoprotection, broilers

## Abstract

**Simple Summary:**

Since the ban on the use of antibiotics as growth promoters in poultry feed, many studies have focused on the use of plants in poultry feed as an alternative to this ban. Currently, many plants are used to improve the growth performance and health status of poultry. Few studies are conducted to evaluate the hepatoprotective effects of these plants in poultry. The current study showed that *Vernonia amygdalina* extract confers a hepatoprotective effect on poultry.

**Abstract:**

The aim of this study was to evaluate the effect of *Vernonia amygdalina* leaf extract (VALE) on the carbon tetrachloride-induced hepatotoxicity (CCl_4_) in broiler chickens. A total of 360-day-old broilers were divided into 4 treatments of 90 birds each consisting of 6 replicates of 15 birds each. The treatments were birds offered 1 mL/kg BW saline (control group), 100 mg/kg BW VALE, 1 mL/kg BW CCl_4_ (CCl_4_-treated group), and 100 mg/kg BW VALE + 1 mL/kg BW CCl_4_ (VALE + CCl_4_ group). Blood samples were collected at 42 days of age and analyzed for the liver enzymes: alanine aminotransferase (ALT), aspartate aminotransferase (AST), alkaline phosphatase (ALP), and selected biochemical parameters. The experiment was laid out in a completely randomized design. The results obtained showed that VALE had the potential to mitigate the adverse effects of CCl_4_ on protein and lipid metabolism as reflected in the low serum malondialdehyde (MDA) levels, which is a marker of lipid peroxidation. The aqueous extract of *Vernonia amygdalina* (VA) at a dose of 100 mg/kg body weight showed a moderate hepatoprotective effect by reducing serum AST levels (*p* < 0.05). The levels of serum AST, ALP, ALT, and GGT were significantly increased in CCl_4_-treated birds compared to the control group, reflecting carbon tetrachloride-induced liver damage. The VALE + CCl_4_ group showed a significantly higher amount of ALP compared to birds treated with carbon tetrachloride, suggesting a hepatoprotective effect. To conclude, *Vernonia amygdalina* aqueous extract can be used to confer protection against hepatotoxicity, which can induce severe hepatocellular damage in birds.

## 1. Introduction

Genetic selection and improvement of economically important traits in broiler production have been used for several years as vital strategies in the commercialization of the poultry industry to increase market body mass and growth rate. This technique has led to the production of fast-growing commercial broilers having altered physiological requirements and that are potentially susceptible to oxidative stress [1]. Oxidative stress is caused by exposure to reactive oxygen intermediates, such as the superoxide anion, hydrogen peroxide, and hydroxyl radical, which can damage proteins, nucleic acids, and cell membranes [2]. Consequently, oxidative stress leads to the initiation and progression of liver damage. 

Carbon tetrachloride (CCl_4_) intoxication has long been known as a model for the induction of toxicity and has been the subject of many toxicological studies in vitro and in vivo [3,4]. The liver is the main target organ for CCl_4_ toxicity due to its high content of cytochrome *p*-450 [5]. It is well established that exposure to CCl_4_ leads to necrosis and cirrhosis of liver tissue induced by the trichloromethyl radical (CCl_3_) activated by cytochrome P450. Necrosis and cirrhosis are reflected by disruption of hepatic metabolic activities and leakage of liver enzymes into the bloodstream [6]. In addition, deleterious effects of CCl_4_ on hepatic antioxidant enzymes, such as catalase (CAT), glutathione peroxidase (GPx), and superoxide dismutase (SOD), have also been reported [7]. Medicinal plants have been shown to be beneficial in ameliorating oxidative damage [8], through elimination of hydrogen peroxide and the scavenging of other free radicals.

*Vernonia amygdalina* (VA) is a shrub of between 1 and 5 m in height, which grows throughout tropical Africa. The plant, generally known as bitter leaf due to the bitterness of its leaves, is used as the main vegetable or spice in the popular bitter-leaf soup [9]. The wood, usually from the root, has been used as a chewing stick. It is much valued as a tooth cleaner, an appetizer, and for gastro-intestinal infection [10]. Some wild chimpanzees have been observed to use this plant for the treatment of parasite-related diseases in Tanzania [11]. The plant is used in traditional medicine for the treatment of a variety of ailments, including malaria [12]. An antidiabetic effect of VA was reported by Erukainure et al. [13], while Okunlola et al. [14] gave a report of its antioxidant properties. Additionally, its anticancer property has been reported [15]. Some bioactive constituents have been identified in the leaves of the plant, including steroid glycosides like vernonioside A1, A2, A3 [16] and vernonioside B2, B3, B4 [17]. Moreover, flavonoids, such as luteolin, luteolin 7-O- glycosides, and luteolin 7-O-glucuronide, as well as certain sesquiterpene lactones [18] have also been reported.

Although there are substantial reports on the nutritional importance of VA [9,19] in poultry feed, there is a paucity of information on its hepatoprotective effects and mechanisms of action in this species. This study was therefore designed to investigate the potential protective effects of VALE on oxidative status and liver damage in broilers subjected to CCl_4_-induced oxidative stress.

## 2. Materials and Methods

### 2.1. Experimental Site

The experiment was carried out at the poultry experimental unit of *Centre d’Excellence Régional sur les Sciences Aviaires* (CERSA) of Université de Lomé, Togo. The experiment was conducted over 6 weeks. Birds were housed in an open-sided deep litter house with a natural environment, ventilation, temperature, and humidity. The average ambient temperature and relative humidity (RH) were 32 ± 5 °C and 45 ± 4%, respectively. The lighting regimen was 23:1 light-dark cycle. The litter materials (wood shavings) were changed every week.

### 2.2. Plant Material and Aqueous Leaf Extract Preparation

Fresh *Vernonia amygdalina* leaf was harvested at Adéticopé, a farm located 30 km north of Lomé (Togo). It was dried in a fresh room at 18 °C and pulverized into powder. Then, a 200-g portion of the powdered leaf was weighed out and dissolved in 2 L of distilled water. The mixture was shaken and kept in the laboratory bench for 24 h before filtering. The extract obtained after filtration was evaporated using rotavapor at 40 °C. The extract was stored at +4 ± 2 °C before use.

### 2.3. Birds, Diets, and Experimental Design

A two-phase feeding program was used, with a starter diet for the first 21 days and a finisher diet from 22 to 42 days of age. The composition of the basal diet is shown in Table 1. Diets were formulated to meet NRC [20] recommendations. A total of 360 broiler chicks (Cobb) were obtained from CERSA hatchery. The experiment was performed as a completely randomized design. The birds were assigned to four treatments of 90 birds each, having 6 replicates of 15 birds each with a stocking density of 30 kg/m^2^. The treatment groups were birds offered 1 mL/kg BW saline (control group), 100 mg/kg BW of *Vernonia amygdalina* leaf extract (VALE), 1 mL/kg BW CCl_4_, and combination of VALE + CCl_4_. From day 21 of the experiment, birds of the control group received isotonic saline (i.p.), whilst experimental groups were administered VALE (orally), and a solution of CCl_4_ in olive oil at a ratio of 1:1 (i.p.). VALE was fed directly into the crop of the birds via a syringe equipped with a plastic nozzle and feeding tube (Nova Cath^®^, No. 10) [21]. Intra-peritoneal injection of CCl_4_ was performed at day 22, and again every three days [22]. In order to homogenize the stress, the control group received 1 mL/kg BW 0.9% sodium chloride solution via intraperitoneal injection [23] and also 2 mL/kg BW of distilled water by oral gavage. Birds had free access to feed and water throughout the experiment. The birds were vaccinated against Gumboro disease (on day 4) and against Newcastle disease and infectious bronchitis (on day 5). A booster of these three vaccines was given after two weeks. The vaccines were administered orally.

### 2.4. Sampling Procedures 

At the onset of the experiment, the initial body weights of the birds were measured and recorded. Feed intake and body weight were measured weekly. Weight gain, average daily weight gain, feed intake, average daily feed intake, and the feed conversion ratio was calculated. CCl_4_ is one of the major hepatotoxins; therefore, the effects attenuated by VALE on CCl_4_-induced liver injury were studied by evaluating the growth performance and biochemical and oxidative parameters. At the end of the trial (42 days), the chicks in each experimental group were weighed individually. Two birds per replicate (12 birds per experimental group; *n* = 12) with average body weight were randomly selected and euthanized by cervical dislocation for blood collection. Blood samples (5 mL) obtained by cardiac puncture were collected into a 10-mL anticoagulant-free vacuum tube and then centrifuged (3000× *g*, 15 min, 4 °C) to obtain serum. The serum obtained was kept on ice and protected from light to avoid oxidation during sampling and stored at −20 °C for biochemical analysis. 

### 2.5. Samples Processing 

The EPPENDORF spectrophotometer (175 Freshwater Blvd Enfield, CT 06082, USA) was used to assay for total serum protein, albumin, total cholesterol, triglycerides, and high-density lipoprotein (HDL) cholesterol using colorimetric methods from specific reagent kits. LDL was calculated using Fredrickson et al.’s [24] formula:

LDL = Total cholesterol − [HDL + (TG/5)]

Enzymatic activities of lactate dehydrogenase (LDH), total antioxidant capacity (TAC), as well as serum content of malondialdehyde (MDA) as antioxidant biomarkers were determined spectrophotometrically, using commercial kits (MyBioSource, Inc. (San Diego, CA 92195-3308 USA)) according to the instructions of the manufacturers. LDH activity was measured at a wavelength of 365 nm using an automated spectrophotometer. TAC was determined using the iron-reducing antioxidant method [25]. The serum content of MDA, an index of lipid peroxidation, was measured by the colorimetric method based on the reaction of 2-thiobarbituric acid (2-TBA) at a wavelength of 532 nm [26]. The serum activities of hepatic alkaline phosphatase (ALP), aspartate aminotransaminase (AST), alanine aminotransferase (ALT), and gammaglutamyl transferase (GGT) as indicators of liver damage were measured using commercial kits purchased from MyBioSource, Inc. (San Diego, CA 92195-3308, USA).

Catalase (CAT) activity was determined according to the method of Sinha [27] while superoxide dismutase (SOD) activity was determined according to the method of Misra and Fridovich [28]. 

### 2.6. Statistical Analysis

The data on growth performance, blood serum biochemical, oxidative biomarkers, and hepatic enzymes were subjected to one-way analysis of variance (ANOVA) using R Software version 4.0.2. When differences among the treatments were significant (*p* < 0.05), means were separated using Tukey’s post hoc test.

## 3. Results

### 3.1. Growth Performance

The effects of VALE and CCl_4_ on the growth performance of broilers at 42 days of experience are presented in Table 2. VALE significantly (*p* < 0.05) improved body weight. Feed intake was significantly (*p* < 0.05) reduced by 3% in the VALE-treated group compared to the control. CCl_4_ significantly (*p* < 0.05) reduced body weight gain (BWG) and feed intake (FI). VALE positively affected FCR.

### 3.2. Oxidative Status

Results for the markers of oxidative stress are shown in Table 3. MDA values in CCl_4_-treated birds were significantly (*p* < 0.05) higher than those of birds in the control group, indicating the oxidative effects of CCl_4_. The combination of VALE + CCl_4_ showed a protective activity of the extract against CCl_4_-mediated lipid peroxidation. CCl_4_-treated birds had a higher serum LDH and lower TAC compared to the control group, indicating an antioxidant suppressive effect of CCl_4_. 

### 3.3. Antioxidant Enzymes

A significant decrease in SOD and CAT activity was observed in the birds treated with CCl_4_ compared to the control group (2130 U/g Hb to 2850 U/g Hb for SOD and 1124 U/g Hb to 935 U/g Hb for CAT, *p* < 0.05, Table 4). VALE significantly increased the activity of SOD and CAT, indicating an hepatoprotective effect of VALE against CCl_4_-induced hepatotoxicity.

### 3.4. Blood Biochemistry

The effects of *Vernonia amygdalina* extract and CCl_4_ and their combination on the blood serum biochemical parameters of broilers are shown in Table 5. In CCl_4_-treated birds, serum albumin and total proteins values decreased by 22% and 47%, respectively, compared to the control group. On the other hand, an increase in protein metabolism activity was observed in CCl_4_ + VALE-treated birds, indicating the ability of the extract to attenuate the deleterious effects of CCl_4_ on protein metabolism. Total cholesterol, triglycerides, and LDL in CCl_4_-treated birds were higher than birds in the control group. This suggests the oxidative effect of CCl_4_ on lipid metabolism. CCl_4_ + VALE-treated birds showed lower levels of triglycerides and LDL than CCl_4_-exposed birds, suggesting a positive alteration in lipid metabolism.

### 3.5. Hepatic Liver Enzyme Activity

The results of the effect of aqueous leaf extract of VALE on CCl_4_-treated birds are shown in Table 6. Birds treated with CCl_4_ showed a higher serum activity of the enzymes ALP, AST, and ALT compared to birds in the control group, reflecting CCl_4_-induced hepatotoxicity. In contrast, birds treated with CCl_4_ + VALE had lower levels of ALP and tended to have lower serum AST levels, suggesting the hepatoprotective effect of the VALE extract against CCl_4_-induced liver damage.

## 4. Discussion

This study evaluated the hepatoprotective effects of *Vernonia amygdalina* in broilers subjected to oxidative stress. In the current study, VALE significantly improved growth performance in terms of BWG and FCR. Birds treated with VALE had better BWG and better FCR. The result from this study is consistent with the findings of Durunna et al. [29] and Tokofai et al. [19], who reported improved growth performance in VA-fed birds. The improvement in weight gain observed in the treated group resulted in a decrease in the feed conversion ratio. The improved performance of the birds may be attributed to the bioactive compounds of VALE. Indeed, it is reported to contain flavonoids, phenolics, alkaloids, terpenes, triterpenoids, steroidal glycosides, sesquiterpene lactones, vitamin A and C, etc. [30,31]. According to the findings of Fiesel et al. [32], supplementation of polyphenol-rich plant products improved the gain:feed ratio in growing pigs. As gut morphology (villus height:crypt depth ratio) and apparent total tract digestibility of nutrients were not generally influenced, the improvement of the gain:feed ratio by the supplementation of the plant products was probably not due to enhanced nutrient digestibility. Similar to our findings, Olobatoke and Oloniruha [33] reported that the inclusion of bitter leaf powder in cockerel diets significantly improved FCR. Adaramoye et al. [34] indicated that the improvement observed may be associated with the beneficial effect of the bitter leaf in strengthening gastrointestinal enzymes, thereby improving digestion and nutrient uptake. Huffman et al. [35] also reported that bitter leaf enhanced the production of gastrointestinal enzymes (chymotrypsin), which may not only improve feed utilization, but also aid digestion of sporozoites and other intestinal parasites affecting feed efficiency.

The liver, being the main target of reactive oxygen species (ROS), is also involved in the response to attacks induced by ROS. Any disturbance in the functioning of liver tissue is expressed through liver biomarkers and enzymatic functions. CCl_4_ is converted to its toxic trichloromethyl free radical by cytochrome P450 2E1 [3]. This free radical, with the potential of oxidative stress, causes destruction of DNA, and disruption of cell structure integrity and hepatic cell metabolism [36]. The better total protein and globulin of the birds of VALE + CCl_4_ indicates the ability of VALE to combat the stress caused by CCl_4_ and modulate the production of proteins by the liver. This ability of VALE to reduce the deleterious effects of CCl_4_ is ensured by its antioxidant compounds, such as phenols and vitamin C. Our observations of the hematochemical parameters in the present study are consistent with the results showing hypoproteinemia and albuminuria in broilers administered CCl_4_ [37], rats [38], and Japanese quail [39]. It is generally thought that oxidative stress is always accompanied by catabolism, as it promotes proteolysis, lipolysis, and glycogenolysis [40]. The elevated serum total cholesterol and triglyceride values in CCl_4_-treated birds in the present study are in agreement with previous studies indicating that oxidative stress reduced the rate of lipolysis and the activity of lipolytic enzymes [41]. However, the cholesterol level in the VALE group was significantly reduced; this may be explained by the presence of cholesterol-lowering compounds in VALE. Saponins have been shown to reduce serum cholesterol levels [42]. Two main mechanisms are involved in the reduction of serum cholesterol by saponins. In the first mechanism, saponins form insoluble complexes with cholesterol, thereby inhibiting its intestinal absorption. The second mechanism suggests that saponins form large aggregates with bile salts in the intestine and thus inhibit ileal reabsorption of bile salts. The latter effect triggers increased synthesis of bile salts from cholesterol in the liver, resulting in depletion of serum cholesterol. The CCl_4_ group birds tended to have lower blood glucose levels compared to the control, contrary to the results of [43]. The main metabolic pathway for the utilization of glucose by skeletal muscles under stress conditions is glycolysis [21], and this can be reflected by the increased activity of LDH under this condition [44]. The significantly lower LDL and significantly higher HDL in the VALE + CCl_4_ group compared to the CCl_4_ group reflects the ability of the bioactive compounds in VALE to mitigate the impact of CCl_4_ stress. It is interesting to note that in addition to the fact that VALE in the present study normalized the HDL of birds subjected to the adverse effect of CCl_4_, this index was even higher than that of the control group. This improvement can be attributed to the pharmacological properties of VALE through its flavonoids [45].

Serum MDA, as well as TAC, ALP, AST, ALT, and GGT are used as indicators of oxidative stress and tissue damage. MDA as an indicator of lipid peroxidation and oxidative stress is used to assess the extent of oxidative degradation of lipids. Birds treated with CCl_4_ in this study had a higher serum MDA content, which reflects lipid peroxidation [46], a result of oxidative stress in biological systems [47]. The binding of the toxic free radical of CCl_4_ (trichloromethyl radical) to macromolecules induces peroxidative degradation of polyunsaturated fatty acids [21]. This degradation leads to the formation of lipid peroxides, which in turn produce MDA with potentially harmful effects [3]. Thus, a lower MDA content indicates a lower reaction of lipid peroxidation [48]. The improved MDA of the birds of VALE + CCl_4_ than those of CCl_4_ in the present study indicates that the bioactive compounds of VALE enhanced the antioxidant status of the birds during exposure to CCl_4_, suggesting its ability to scavenge peroxyl and hydroxyl radicals. Indeed, the antioxidant property of VALE has been attributed to the presence of their flavonoids. Our results differ from those of Moradi et al. [39], who reported that there was no difference in the effect of CCl_4_ on lipid peroxidation in Japanese quail. This discrepancy in the results may be due to the difference in poultry species investigated. The rapid growth rate of the commercial broiler chickens could be responsible for the susceptibility to oxidative stress conditions. Birds in the CCl_4_ + VALE group showed a lower rate of tissue damage in this study. This result suggests the ability of VALE to attenuate the toxic effects of CCl_4_ by reducing the increased activity of serum enzymes. The results obtained are in agreement with those showing a significant increase in serum AST and ALT levels in broilers treated with CCl_4_ [22] and Wistar rats [49], AST in Japanese quails [50], and ALP in Japanese quails [51].

In this study, the elevated serum ALP and GGT concentrations in the CCl_4_ group birds can be attributed to liver damage and biliary system damage. Adesanoye and Farombi [52] reported the hepatoprotective and antioxidant potential of VA (500 mg/kg body weight) in rats. VA could therefore act by inhibiting the activation of CCl_4_ by inactivating the cytochrome P450 system. As a potent antioxidant, it can also influence the enzymatic systems associated with glutathione and superoxide dismutase through the elimination of free radicals. Action on cytochrome P450 or inhibition of oxidative damage may be responsible for the protective effect against liver damage induced by oxidative stress, such as the findings observed in the present study. In agreement with our results, it was demonstrated that the negative effect of CCl_4_ could be attenuated by *Zingiber officinale* [53] and green tea [54] and *Tanacetum parthenium* [43].

Serum CAT and SOD concentrations were significantly lower in the CCl_4_-treated groups compared to those in the VALE group. The enzymatic antioxidant system comprising SOD and CAT is the first line of antioxidant defense that converts harmful molecules, including hydrogen peroxides and hydroperoxides, into harmless molecules [55]. Similarly, SOD catalyzes the excess superoxide radicals into hydrogen peroxide and oxygen; CAT catalyzes the decomposition of hydrogen peroxide into water and molecular oxygen [55]. The better upregulation of SOD and CAT of the birds treated with CCl_4_ and supplemented with VALE than the birds with CCl_4_-induced hepatotoxicity without VALE in this study suggests that improvement in these endogenous enzymes is among the protective mechanisms of action of VALE. This improvement may be attributable to the polyphenols in VALE, which have the ability to reverse the oxidative stress-induced impairment. The results obtained in this study are in agreement with a report where a decrease in the concentration of SOD in broilers [21] was reported. This decrease could be due to the inhibitory effect of MDA on antioxidant enzymes and its accelerating effect on oxidative damage on biomolecules.

## 5. Conclusions

Overall, the results obtained in this study confirmed the hepatoprotective effects of VALE following CCl_4_-induced hepatotoxicity. This study demonstrated that *Vernonia amygdalina* bioactive compounds can mitigate oxidative stress-induced homeostasis disruption in broilers through modulation of oxidative stress biomarkers.

## Figures and Tables

**Table 1:** Composition and calculated analyses of the basal diet ^1^.

Ingredients (%).	0 to 21 Days	22 to 42 Days
White maize	57.00	66.00
Roasted soya bean meal	25.00	24.00
Wheat bran	5.00	3.00
Fish meal 40%	5.00	2.00
Oyster shell	2.00	2.50
Concentrate 2	5.00	2.50
Lysine	0.50	-
Methionine	0.50	-
Calculated analysis		
Metabolizable energy (MJ)	12.7	13.0
Crude protein (%)	21.43	18.45
Crude fiber (%)	4.71	4.74
Lysine (%)	1.37	1.14
Methionine (%)	0.54	0.43
Methionine + Cystine (%)	0.68	0.54
Calcium (%)	1.17	1.13
Phosphorus (%)	0.66	0.50

^1^ Calculated composition was according to NRC [20]. ^2^ Concentrate (mineral and vitamin complex) provided per kilogram of diet: Transretinyl acetate, 60 mg; cholecalciferol, 1.5 mg; α-tocopherol acetate, 400 mg; bisulphate menadione complex, 40mg; thiamine mononitrate, 30 mg; riboflavin, 120 mg; nicotinic acid, 200 mg; Pyridoxine, 100 mg; Cyanocobalamin, 0.4 mg; folic acid, 20 mg; d-biotin, 2 mg; choline chloride, 7000 mg; iron, 36 mg; iodine, 1.6 mg; manganese, 48 mg; zinc, 56 mg; copper, 12 mg; selenium, 0.32 mg.

**Table 2:** Effect of *Vernonia amygdalina* leaf extract and carbon tetrachloride (CCl_4_) on the growth performance of broiler chickens.

Parameters	Treatments					
	Control	VALE	CCl_4_	VALE + CCl_4_	SEM	*p*-Value
Initial body weight (g)	43.25	43.53	44.02	43.01	0.13	0.152
Final body weight (g)	1725 ^b^	1858 ^a^	1647 ^c^	1716 ^b^	4.45	0.003
Weight gain (g)	1682 ^b^	1814 ^a^	1603 ^c^	1673 ^b^	8.57	0.005
Daily weight gain (g)	40.05 ^b^	43.19 ^a^	38.17 ^c^	39.83 ^b^	0.32	<0.0001
Total feed intake (g)	3602 ^a^	3517 ^b^	3505 ^c^	3528 ^b^	7.66	0.014
Daily feed intake (g)	85.75 ^a^	83.73 ^c^	83.45 ^c^	84.01 ^b^	0.71	0.002
Feed conversion ratio	2.14 ^a^	1.93 ^b^	2.12 ^a^	2.11 ^a^	0.02	0.011

^a,b,c^ Means with different superscripts on the same row differ significantly (*p* < 0.05).

**Table 3:** Effect of experiment treatments on blood serum oxidative biomarkers at 42 days of age in broiler chickens.

Parameters	Treatments					
	Control	VALE	CCl_4_	VALE + CCl_4_	SEM	*p*-Value
LDH (U/L)	100.9 ^b^	110.2 ^a,b^	116.7 ^a^	118.3 ^a^	3.60	0.006
MDA (nmol/mL)	1.87 ^c,d^	1.91 ^b,c^	2.75 ^a^	1.96 ^b^	0.16	<0.0001
TAC (U/mL)	1.89 ^a^	1.83 ^a^	1.15 ^b^	1.26 ^b^	0.13	0.043

^a,b,c^ Means with different superscripts on the same row differ significantly (*p* < 0.05). LDH = Lactate dehydrogenase; MDA = Malondialdehyde; TAC = Total antioxidant capacity.

**Table 4:** Effect of experiment treatments on antioxidant enzymes at 42 days of age in broiler chickens.

Parameters	Treatments					
	Control	VALE	CCl_4_	VA + CCl_4_	SEM	*p*-Value
SOD (U/g Hb)	2850 ^b^	3925 ^a^	2130 ^c^	2925 ^b^	100.1	0.034
CAT (U/g Hb)	1124 ^b^	1830 ^a^	935 ^c^	1055 ^b^	62.27	0.022

^a,b,c^ Means with different superscripts on the same row differ significantly (*p* < 0.05). SOD = Superoxide dismutase; CAT = Catalase.

**Table 5:** Effect of experiment treatments on blood serum biochemical parameters at 42 days of age in broiler chickens.

Parameters	Treatments					
	Control	VALE	CCl_4_	VALE + CCl_4_	SEM	*p*-Value
Glucose (mg/dL)	224.3 ^a^	226.9 ^a^	211.07 ^b^	217.6 ^a^	1.34	0.026
Total protein (g/dL)	3.35 ^a^	3.47 ^a^	1.76 ^b^	3.26 ^a^	0.26	0.003
Albumin (g/dL)	1.76 ^a^	1.98 ^a^	1.37 ^b^	1.78 ^a^	0.03	0.010
Total cholesterol (mg/dL)	138.3 ^a^	120.8 ^b^	140.4 ^a^	142.7 ^a^	2.48	0.021
Triglyceride (mg/dL)	43.29 ^a^	34.46 ^b^	49.18 ^a^	42.83 ^a^	1.50	0.009
LDL (mg/dL)	107.8 ^b^	92.18 ^c^	117.5 ^a^	94.31 ^c^	1.40	0.005
HDL (mg/dL)	54.9 ^b^	73.24 ^a^	42.38 ^c^	75.53 ^a^	1.33	0.002

^a,b,c^ Means with different superscripts on the same row differ significantly (*p* < 0.05). LDL = Low-Density Lipoprotein; HDL = High-Density Lipoprotein.

**Table 6:** Effect of experimental treatments on blood serum activity of hepatic enzymes at 42 days of age in broiler chickens.

Parameters	Treatments					
	Control	VALE	CCl_4_	VALE + CCl_4_	SEM	*p*-Value
AST (U/L)	234.1 ^a^	239.7 ^a^	257.9 ^b^	245.3 ^b^	2.43	0.034
ALP (U/L)	221.6 ^a^	246.1 ^b^	282.8 ^c^	255.1 ^b^	3.46	0.002
ALT (U/L)	17.6 ^a^	18.9 ^a^	20.51 ^b^	20.24 ^b^	0.44	0.017
GGT (U/L)	12.35 ^a^	12.95 ^a^	14.7 ^b^	13.32 ^ab^	0.46	0.021

^a,b,c^ Means with different superscripts on the same row differ significantly (*p* < 0.05). AST = Aspartate aminotransferase; ALP = Alkaline phosphatase; ALT = Alanine aminotransferase; GGT = Gamma-glutamyl transferase.

## Data Availability

The data presented in this study are available on request from the corresponding author.
